# Varying diffusion curvature, intravoxel incoherent motion, and conventional monoexponential diffusion models for evaluating Ki-67 expression in rectal carcinoma

**DOI:** 10.3389/fonc.2026.1935475

**Published:** 2026-10-21

**Authors:** Yingying Cui, Huijia Yin, Ying Wang, Yue Liu, Yaping Wu, Yan Bai, Hanhan Li, Baiyan Jiang, Nan Meng, Xuan Yu

**Affiliations:** 1 Department of Radiology, Henan Provincial People’s Hospital and Zhengzhou University People’s Hospital, Zhengzhou, China; 2 Department of MRI, The First Affiliated Hospital of Henan Medical University, Weihui, China; 3 Clinical and Technical Support, Philips Healthcare, Beijing, China

**Keywords:** intravoxel incoherent motion, Ki-67, magnetic resonance imaging, rectal carcinoma, varying diffusion curvature

## Abstract

**Objectives:**

This study aimed to systematically evaluate and compare the diagnostic performance of three diffusion models — varying diffusion curvature (VDC), intravoxel incoherent motion (IVIM), and conventional monoexponential diffusion-weighted imaging (DWI) — in predicting Ki-67 expression status in rectal adenocarcinoma.

**Methods:**

A total of 84 patients with rectal carcinoma were enrolled, comprising 48 cases with high proliferative activity (Ki-67 > 50%) and 36 cases with low proliferative activity (Ki-67 ≤ 50%). All patients underwent pelvic MRI examinations. The following imaging parameters were calculated and compared: VDC-derived parameters (D_0_, D_1_), IVIM-derived parameters (ADC_slow_, ADC_fast_, f), and the DWI-derived parameter (ADC). Logistic regression analysis was performed to identify independent predictive factors, and diagnostic performance was assessed using the area under the receiver operating characteristic curve (AUC), the DeLong test, and calibration curves.

**Results:**

Compared with the low-proliferation group, the high-proliferation group of rectal carcinoma exhibited significantly higher D_1_ and f values, along with markedly lower D_0_, ADC, and ADC_slow_ values (all P < 0.05). Logistic regression analysis identified D_1_ and ADC_slow_ as independent predictors of Ki-67 status in rectal carcinoma. The combined diagnostic model incorporating D_1_ and ADC_slow_ yielded an AUC of 0.924, which was significantly superior to the individual parameters, including ADC (AUC = 0.780), ADC_slow_ (AUC = 0.832), D_0_ (AUC = 0.696), D_1_ (AUC = 0.857), and f (AUC = 0.802) (all P < 0.05). The calibration curve demonstrated good stability and reliability of the combined diagnostic model.

**Conclusion:**

Both VDC, IVIM, and DWI demonstrate efficacy in evaluating the Ki-67 expression status in rectal carcinoma. The combined parameters of D_1_ and ADC_slow_ may serve as a novel and reliable imaging biomarker for assessing the proliferative activity of rectal carcinoma.

## Introduction

Colorectal carcinoma represents one of the most prevalent malignancies of the digestive system, and its global incidence and mortality have shown a sustained upward trend in recent years, imposing a substantial burden on healthcare systems worldwide (1, 2). Unregulated cellular proliferation is a core hallmark of malignant neoplasms, and the proliferative activity of tumor cells is closely associated with disease progression, treatment response, and patient prognosis. As a nuclear phosphoprotein expressed throughout all active phases of the cell cycle, Ki-67 has been widely recognized as a reliable immunohistochemical marker for quantifying tumor proliferative capacity and biological aggressiveness (3). Previous meta- analyses have consistently indicated that elevated Ki-67 expression in rectal carcinoma is correlated with higher tumor grade, increased risk of recurrence, and inferior long-term survival outcomes (4, 5). At present, pathological biopsy is the clinical gold standard for determining Ki-67 expression status. Nevertheless, biopsy is an invasive procedure, and its diagnostic accuracy may be compromised by sampling bias, intratumoral heterogeneity, and operator-dependent variability. Hence, there is an urgent clinical demand for a noninvasive, reproducible approach to preoperatively evaluate Ki-67 expression in patients with rectal carcinoma.

Magnetic resonance imaging (MRI), as a radiation-free, noninvasive imaging modality, plays an indispensable role in the preoperative staging, treatment response assessment, and follow-up surveillance of rectal carcinoma (6). However, conventional morphological MRI sequences primarily provide macroscopic information regarding tumor size, shape, and local invasion, which cannot accurately reflect the microscopic cellular and molecular characteristics of lesions. As a functional MRI technique, diffusion-weighted imaging (DWI) probes the random thermal motion of water molecules in biological tissues via multi-b-value gradient encoding (7). When fitted with the simplest monoexponential model, it yields the apparent diffusion coefficient (ADC), a quantitative parameter that has been confirmed in multiple systematic reviews to be associated with Ki-67 expression levels in rectal carcinoma (8, 9). Despite its widespread clinical application, the conventional monoexponential DWI model is built upon the assumption of Gaussian water diffusion, which fails to account for the complex microenvironment of tumor tissues. The measured ADC values are easily confounded by tissue microcirculatory perfusion, and this simplified model cannot fully characterize the restricted diffusion behavior at the cellular level, thus limiting its diagnostic accuracy for tumor phenotyping.

The intravoxel incoherent motion (IVIM) model is an advanced bi-exponential diffusion signal fitting approach that can be applied to multi-b-value DWI data, which decouples and separately quantifies the true diffusion of water molecules and the pseudo-diffusion effect caused by microcirculatory perfusion from the same acquisition (10). Accumulating evidence has demonstrated the utility of IVIM-derived parameters for evaluating Ki-67 expression in multiple tumor types, including breast cancer, soft tissue sarcoma, and non-small cell lung cancer (11–13). However, existing research on the association between IVIM metrics and Ki-67 in colorectal cancer remains limited, with most studies featuring small sample sizes and inconsistent conclusions (14, 15). The varying diffusion curvature (VDC) model is an emerging non-Gaussian diffusion signal decay model that describes the b-value-dependent attenuation of the diffusion signal with higher precision (16). The core principle of VDC is that the apparent diffusion coefficient of tissue does not remain constant but decreases progressively with increasing b-value, especially in the high b-value range. Through fitting the signal attenuation curve, the VDC model yields two independent parameters that reflect tissue cellular density and microstructural complexity, respectively. To date, the clinical application of the VDC model has only been reported in hepatocellular carcinoma for the prediction of microvascular invasion (17), and its diagnostic value for rectal carcinoma proliferative assessment remains unexplored.

Therefore, this study aims to comprehensively evaluate and compare the performance of three diffusion fitting models (conventional monoexponential DWI, IVIM, and VDC) in assessing the proliferative status of rectal carcinoma, identify independent predictive factors, and determine the optimal combination of diagnostic parameters, thereby offering a novel reference for clinical diagnosis and therapeutic decision-making.

## Materials and methods

### Study population

This retrospective observational diagnostic accuracy study was approved by the Institutional Review Board of our hospital, and all enrolled participants provided written informed consent before MRI examination, in accordance with the Declaration of Helsinki. We consecutively screened 137 patients with clinically suspected rectal carcinoma admitted to our hospital between April 2023 and March 2026. Patients were excluded based on the following criteria: (1) 11 individuals who did not complete the full MRI examination protocol; (2) 9 cases with MRI images of insufficient quality for quantitative post-processing; (3) 12 patients who had received any antitumor therapy prior to MRI scanning; (4) 8 subjects with an interval exceeding 14 days between MRI acquisition and pathological biopsy; (5) 5 cases with indeterminate or non-diagnostic pathological findings; and (6) 9 patients with final pathological diagnoses other than rectal carcinoma. After applying these exclusion criteria, 84 patients were ultimately included in this retrospective analysis (**Figure 1**). The study protocol was reviewed and approved by the institutional review board of our hospital, and written informed consent was obtained from all participants prior to enrollment. Baseline clinical characteristics, including age, sex, maximum tumor diameter, body mass index (BMI), and serum carcinoembryonic antigen (CEA) levels, were collected and analyzed.

**Figure 1:**
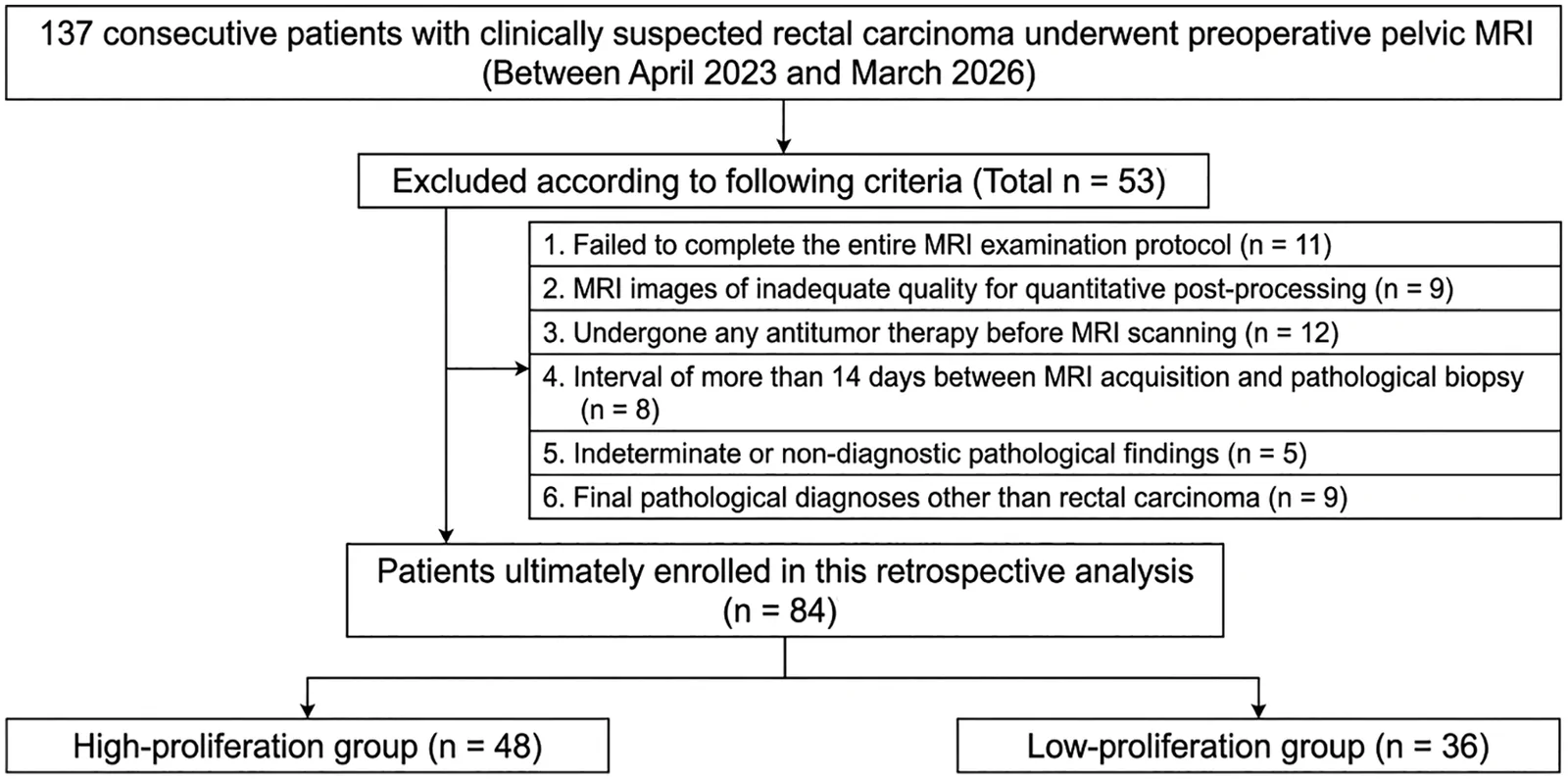
Flowchart illustrating the patient selection process.

### Image acquisition

All MRI examinations were performed on a 3.0T whole-body MRI system (Philips Healthcare, Best, the Netherlands) using a 16-channel phased-array body coil for signal reception. Patients were placed in the supine position and scanned in a feet-first orientation. The scanning protocol included axial two-dimensional fast spin-echo T2-weighted imaging (T2WI) and multi-b-value single-shot echo-planar diffusion-weighted imaging (EPI-DWI) sequences. The detailed parameters of the T2WI sequence were set as follows: repetition time (TR) = 4500 ms, echo time (TE) = 120 ms, field of view (FOV) = 22 × 22 cm, matrix size = 288 × 320, bandwidth = 62.50 Hz per pixel, slice thickness = 3 mm, inter-slice gap = 0.3 mm, and total acquisition time = 149 s. For the multi-b-value DWI sequence, diffusion-sensitizing gradients were applied along three orthogonal directions with b-values of b = 0, 25, 50, 100, 150, 200, 400, 600, 800, 1000, 1500, and 2000 s/mm^2^ s/mm². Other sequence parameters included: TR = 4400 ms, TE = 89.2 ms, FOV = 34 × 34 cm, matrix size = 128 × 128, bandwidth = 250 Hz per pixel, slice thickness = 3 mm, and inter-slice gap = 0.3 mm, with a total scanning time of 277 s. To improve scanning efficiency and ensure spatial consistency, the multi-b-value DWI sequence was positioned with reference to the axial T2WI images, covering all slices containing the primary tumor, with consistent slice positions, thickness, and gaps across both sequences.

### Parameter generation

All quantitative parameters were derived from a single set of raw multi-b-value DWI data, which were exported and imported into MATLAB R2018b software (MathWorks Inc., Natick, MA, USA) for quantitative post-processing. Before parameter fitting, uniform preprocessing steps including B0 field inhomogeneity correction, gradient nonlinearity correction, and eddy current distortion correction were performed on the original data to reduce systematic errors. Three distinct diffusion signal decay models were then applied to the parametric map generation, with corresponding Equations 1–3 as follows:

(1) $$S_{b}/S_{0}=\;exp\;\left(-\;b\;\times \;ADC\right)$$

(2) $$S_{b}/S_{0}=\;\left(1\;-\;f\right)\;\times \;exp\left(-bADC_{slow}\right)\;+\;f\;\times \;exp\;\left[-b\times \left(ADC_{fast}+ADC_{slow}\right)\right]$$

(3) $$S_{b}/S_{0}=\exp\left[-\left(D_{0}/D_{1}\right)\left(1-\exp\left(-b\times D_{1}\right)\right)\right]$$

In the above formulas, b refers to the diffusion-sensitizing gradient factor, S_0_ represents the signal intensity without diffusion weighting (b=0 s/mm²), and S_b_ denotes the signal intensity at a given b-value. For the conventional DWI model, ADC is the apparent diffusion coefficient reflecting overall water molecule mobility (18). For the IVIM model, ADC_slow_ represents the true diffusion coefficient of water molecules in the tissue interstitium (corresponding to D in standard IVIM nomenclature), ADC_fast_ is the pseudo-diffusion coefficient related to microcirculatory blood flow (corresponding to D*) in standard IVIM nomenclature), and f is the perfusion fraction reflecting the proportion of microvascular perfusion in the total signal attenuation (19). For the VDC model, D_0_ is a diffusion coefficient associated with tissue cellular density, and D_1_ characterizes the degree of water diffusion restriction, which indirectly reflects the microstructural complexity of tumor tissue (17, 20).

In line with the respective theoretical principles and optimal fitting ranges of each diffusion model, three tailored b-value subsets were selected for signal fitting: the conventional mono-exponential ADC was estimated using the standard clinical two-point method with b = 0 and b = 800 s/mm²; IVIM modeling was performed using b-values of 0, 25, 50, 100, 150, 200, 400, 600, 800, and 1000 s/mm² to adequately capture both microcirculatory perfusion effects at low b-values and true tissue water diffusion at intermediate b-values; VDC fitting was conducted using b-values of 0, 50, 100, 200, 600, 800, 1500, and 2000 s/mm² to optimally characterize the curvature of signal decay across a wide high-b-value range. Voxel-wise nonlinear fitting for IVIM and VDC was performed using the Levenberg–Marquardt optimizer with equal weighting of all included b-value points; convergence was defined as a relative change in the sum of squared residuals below 1×10^-6^, with a maximum of 1000 iterations. Physiologically constrained initial values and parameter bounds were set for each nonlinear model. A voxel was classified as a failed fit if any parameter exceeded its predefined bounds, if the optimizer failed to converge within 1000 iterations, or if the residual sum of squares was greater than three times the noise variance; such voxels were excluded from subsequent analysis. Across the entire cohort, failed voxels within tumor accounted for less than 2% of all voxels for every parameter, which was considered negligible.

All multi-b-value DWI images were first rigidly coregistered to the corresponding axial T2WI using the b=0 s/mm² image as the reference, to eliminate spatial mismatch between sequences. On each axial slice, region of interests (ROIs) were manually delineated layer by layer along the entire contour of the solid tumor component, generating a three-dimensional volume of interest (VOI) that covered the full volume of the primary lesion. With side-by-side reference to T2WI and high-b-value (1000 s/mm²) DWI, the following structures were carefully excluded from the VOI to avoid measurement bias: (1) intratumoral cystic degeneration, coagulative necrosis, hemorrhage, and dystrophic calcification; (2) adjacent normal rectal mucosa and muscularis propria; (3) intraluminal gas, fecal contents, and perirectal adipose tissue, to minimize partial volume effects. The finalized 3D VOIs were then affine-registered and mapped to each of the six parametric maps (ADC, ADC_slow_, ADC_fast_, f, D_0_, D_1_) using an in-house MATLAB algorithm, ensuring identical spatial positioning across all quantitative maps. The mean value of each parameter within the entire VOI volume was automatically extracted and exported for subsequent statistical analysis. Above works were independently completed by two radiologists with 6 and 9 years of abdominal MRI experience, respectively, both of whom were blinded to all clinical and pathological information of the patients.

### Ki-67 analyses

Ki-67 expression status was determined via immunohistochemical staining using a mouse anti-human Ki-67 monoclonal antibody (clone MIB-1, DAKO, Glostrup, Denmark) on postoperative pathological specimens. All pathological slides were independently evaluated by two senior pathologists who were blinded to the imaging results. Under a light microscope at 400× magnification, five representative fields with the highest density of Ki-67-positive tumor cells (hotspot areas) were selected for each specimen. The total number of tumor cells and the number of positively stained nuclei in each field were counted, and the Ki-67 labeling index was calculated as the percentage of positively stained tumor cells. In accordance with previous clinical classification criteria (21, 22), patients were divided into two groups based on Ki-67 expression level: the low-proliferation group included cases with weak positive (+, Ki-67 <10%) or moderate positive (++, Ki-67 10%–50%) expression, and the high-proliferation group included cases with strong positive (+++, Ki-67 >50%) expression.

### Statistical analysis

All statistical analyses were performed using MedCalc software (version 15.0; MedCalc Software, Ostend, Belgium), and a two-tailed P-value < 0.05 was considered statistically significant. Interobserver agreement was quantified using intraclass correlation coefficients (ICCs) calculated with a two-way mixed-effects model with absolute agreement definition. Average-measures ICC (representing the reliability of the mean value from the two readers) was computed. An ICC value greater than 0.75 was defined as indicating excellent interobserver reliability (23). The normality of continuous variables was tested using the Shapiro-Wilk test. Normally distributed continuous data were expressed as mean ± standard deviation, and intergroup differences were compared using the independent samples t-test. Non-normally distributed continuous variables were presented as median (interquartile range) and compared using the Mann-Whitney U test. Categorical variables were described as counts (percentages) and analyzed using the chi-square test or Fisher’s exact test as appropriate. Receiver operating characteristic (ROC) curve analysis was performed to evaluate the diagnostic efficacy of each imaging parameter and the combined model for identifying high-proliferation rectal carcinoma, with the area under the ROC curve (AUC) as the main evaluation metric. The DeLong test was used to compare differences in AUC values between different diagnostic models. Univariate and multivariate logistic regression analyses were conducted to screen for independent predictors of high Ki-67 expression. The variance inflation factor (VIF) is used to assess multicollinearity, and a VIF value of less than 5 is considered to indicate the absence of multicollinearity. A nomogram was constructed to visually present the predictive contribution of each independent factor, and calibration curve analysis was performed to verify the consistency between the predicted probability and the actual probability of the combined model. Bootstrap (1,000 resamples) is used for internal validation of the combined model.

## Results

### Baseline data

A total of 84 patients with pathologically confirmed rectal carcinoma were included in the final analysis, aged 24 to 84 years (52 males, 32 females). Among all patients, 48 were assigned to the high-proliferation group and 36 to the low-proliferation group. Intergroup comparison showed no statistically significant differences in age, sex, BMI, maximum tumor diameter, or serum CEA level between the high- and low-proliferation groups (all P > 0.05, **Table 1**).

**Table 1:** Comparison of different variables between high- and low-proliferation group.

Variables	Low-proliferation (n = 36)	High-proliferation (n = 48)	t/χ^2/^z value	P-value
Age (years)	58.86 ± 11.27	62.21 ± 9.20	1.445	0.150^a^
Maximum diameter (cm)	4.25 (3.23, 5.20)	3.70 (2.93, 4.50)	-1.407	0.159^b^
Sex, n (%)			2.844	0.092^b^
Male	26 (72.22%)	26 (54.17%)		
Female	10 (27.78%)	22 (45.83%)		
BMI	24.02 (22.26, 26.61)	24.27 (22.43, 27.33)	-0.217	0.828^b^
CEA (ng/ml)	3.01 (1.34, 8.25)	2.81 (1.56, 7.04)	-0.018	0.986^c^
ADC (×10^-3^mm^2^/s)	1.40 (1.20, 1.78)	1.05 (0.90, 1.28)	-4.440	< 0.001^b^
D_0_ (×10^-3^mm^2^/s)	1.50 (1.25, 1.76)	1.22 (1.12, 1.42)	-3.069	0.002^b^
D_1_	0.44 ± 0.09	0.63 ± 0.14	6.822	< 0.001^a^
ADC_slow_	1.14 ± 0.22	0.87 ± 0.18	-6.080	< 0.001^a^
f	19.62 ± 4.22	24.60 ± 4.53	5.185	< 0.001^a^
ADC_fast_	60.93 ± 9.87	63.89 ± 11.28	1.227	0.205^a^

^a^ Independent t-test; ^b^Chi-squared test; ^c^Mann–Whitney U test.

### Consistency evaluation

Interobserver agreement for the parameters ADC, ADC_slow_, ADC_fast_, f, D_0_, and D_1_ was excellent, with all ICCs were 0.936 (95% CI: 0.903 - 0.958), 0.925 (95% CI: 0.887 - 0.951), 0.851 (95% CI: 0.780 - 0.901),0.895 (95% CI: 0.842 - 0.931),0.933 (95% CI: 0.899 - 0.956), 0.914 (95% CI: 0.871 - 0.944), respectively. The mean values obtained from the two assessors were used for subsequent analyses.

### Parameter comparison

Compared with the low-proliferation group, the high-proliferation group exhibited significantly higher D_1_ and f values, and significantly lower D_0_, ADC, and ADC_slow_ values (all P < 0.05). No significant difference was found in ADC_fast_ values between the two groups (P = 0.205; see **Table 1**; **Figures 2**, **3**).

**Figure 2:**
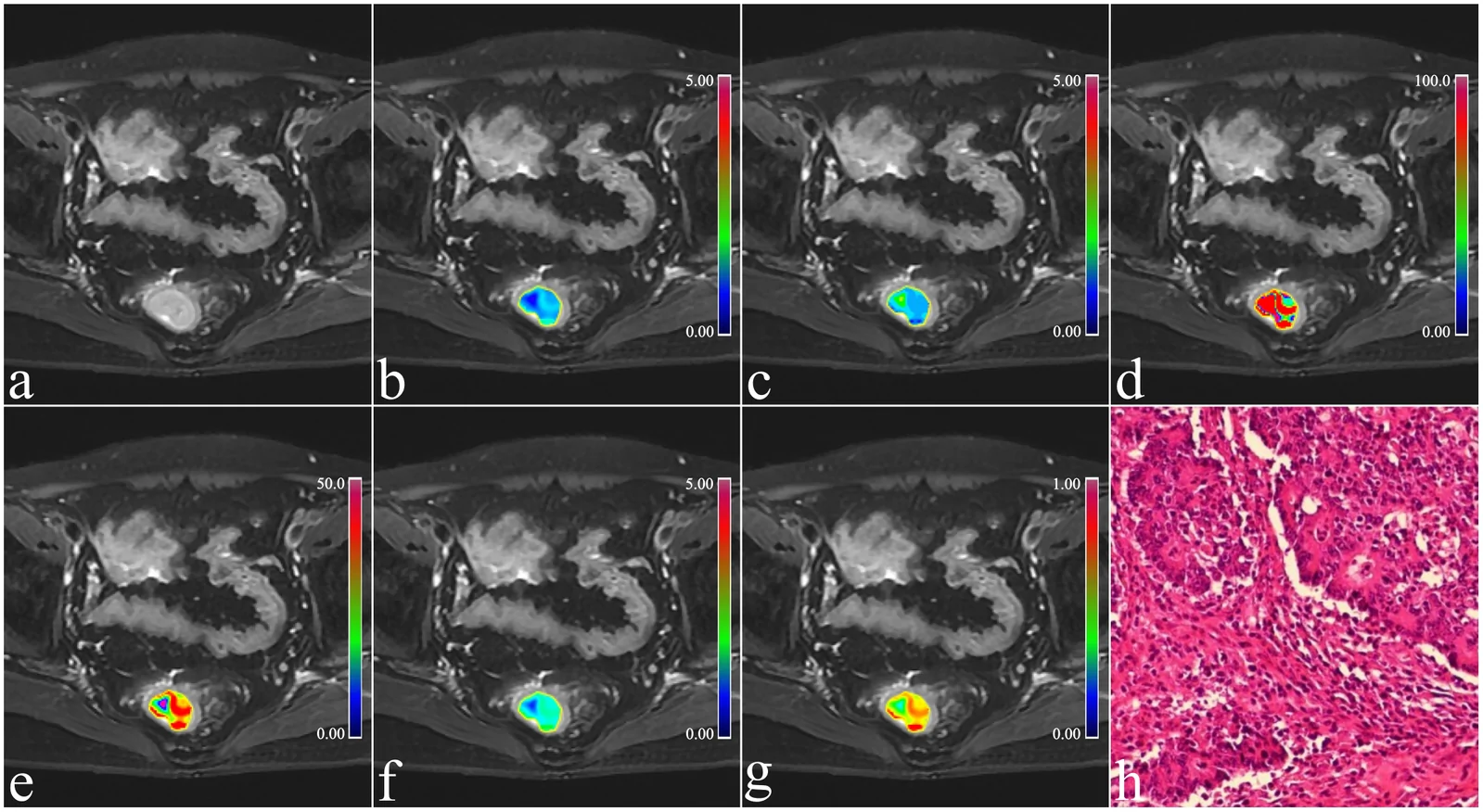
A 57-year-old female patient diagnosed with rectal carcinoma (maximum tumor diameter = 3.2 cm, CEA level = 2.43 ng/ml, Ki-67 = 80%). **(a)** T2-weighted image; **(b)** Fusion image of ADC pseudo-color map with T2WI; **(c)** Fusion image of ADC_slow_ pseudo-color map with T2WI; **(d)** Fusion image of ADC_fast_ pseudo-color map with T2WI; **(e)** Fusion image of f pseudo-color map with T2WI; **(f)** Fusion image of D_0_ pseudo-color map with T2WI; **(g)** Fusion image of D_1_ pseudo-color map with T2WI; **(h)** Pathological images (original magnification, ×100).

**Figure 3:**
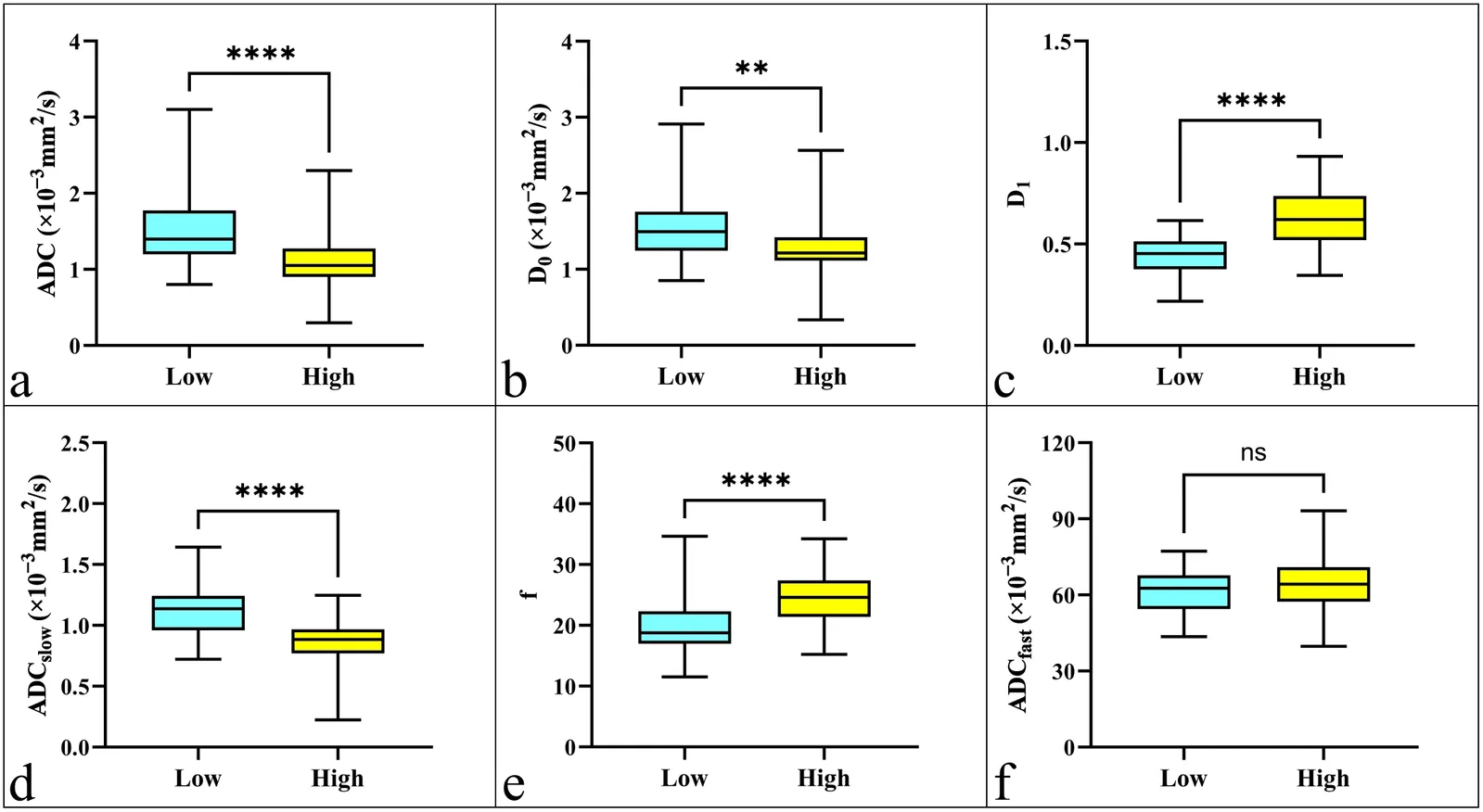
Box plots comparing parameter differences between low- and high-proliferation rectal carcinoma groups: **(a)** ADC; **(b)** D_0_; **(c)** D_1_; **(d)** ADC_slow_; **(e)** f; **(f)** ADC_fast_. **** P < 0.0001, **P < 0.01.

### Independent predictors

All baseline clinical variables (age, sex, BMI, maximum tumor diameter, CEA) and imaging quantitative parameters (ADC, ADC_slow_, ADC_fast_, f, D_0_, D_1_) were included in the logistic regression analysis. Univariate logistic regression showed that among all included variables, only the five imaging parameters (ADC, ADC_slow_, f, D_0_, D_1_) were significantly associated with high Ki-67 expression (all P < 0.05). Variables with statistical significance in univariate analysis were further included in multivariate logistic regression. The results indicated that ADC_slow_ and D_1_ were independent predictive factors for high proliferative activity in rectal carcinoma, with P values of 0.022 and <0.001, respectively (**Table 2**).

**Table 2:** Univariate and multivariate logistic regression analyses.

Variables	Univariate analyses				Multivariate analyses		
	OR*-Value	95% CI	P-Value	VIF	OR*-Value	95% CI	P-Value
Age (year)	1.403	0.893 - 2.205	0.142	1.156	/	/	/
Maximum diameter (mm)	0.780	0.502 - 1.213	0.270	1.137	/	/	/
Sex	0.680	0.433 - 1.069	0.095	1.106	/	/	/
BMI	1.122	0.714 - 1.763	0.619	1.263	/	/	/
CEA (ng/ml)	0.631	0.297 - 1.342	0.232	1.155	/	/	/
ADC (×10^-3^mm^2^/s)	0.316	0.163 - 0.611	< 0.001	3.588	0.588	0.171 - 2.021	0.400
D_0_ (×10^-3^mm^2^/s)	0.499	0.298 - 0.837	0.008	2.889	1.100	0.364 - 3.324	0.865
D_1_	8.437	3.255 - 21.870	< 0.001	1.953	9.347	2.576 - 33.915	< 0.001
ADC_slow_	0.165	0.075 - 0.366	< 0.001	3.040	0.228	0.064 - 0.808	0.022
f	3.906	2.012 - 7.583	< 0.001	1.511	1.879	0.808 - 4.371	0.143
ADC_fast_	1.329	0.849 - 2.079	0.213	1.631	/	/	/

OR, odds ratio; CI, confidence interval; VIF, variance inflation factor; * OR for per 1 standard deviation.

### Diagnostic performance

The integrated diagnostic model incorporating the independent predictors ADC_slow_ and D_1_ achieved an AUC of 0.924, with a sensitivity of 70.83% and specificity of 97.22%. This performance was significantly superior to that of individual parameters, including ADC (AUC = 0.780), ADC_slow_ (AUC = 0.832), f (AUC = 0.802), D_0_ (AUC = 0.696), and D_1_ (AUC = 0.857), as indicated by Z values of 3.013, 2.589, 2.407, 3.771, and 2.051, respectively (all P < 0.05).

### Internal validation and calibration curves

The AUC for internal validation of the combined model based on Bootstrap was 0.917(95% CI: 0.904 - 0.923). Calibration curve analysis further confirmed the model’s strong stability and reliability (**Table 3**; **Figures 4**, **5**).

**Table 3:** AUCs of different variables.

Variables	AUC	95% CI	Cutoff	Specificity	Sensitivity	Comparison with combined diagnosis
ADC (×10^-3^mm^2^/s)	0.780	0.676 - 0.863	1.100	83.33%	70.83%	Z = 3.013, P = 0.003
D_0_ (×10^-3^mm^2^/s)	0.696	0.586 - 0.792	1.423	63.89%	77.08%	Z = 3.771, P < 0.001
D_1_	0.857	0.764 - 0.924	0.513	77.78%	81.25%	Z = 2.051, P = 0.040
ADC_slow_	0.832	0.735 - 0.905	1.002	75.00%	83.33%	Z = 2.589, P = 0.010
f	0.802	0.701 - 0.881	22.77	86.11%	68.75%	Z = 2.407, P = 0.016
ADC_fast_	0.569	0.456 - 0.677	55.64	33.33%	85.42%	Z = 5.490, P < 0.001
Combined Diagnosis	0.924	0.845 - 0.971	0.793	97.22%	70.83%	/

Combined Diagnosis, ADC_slow_ + D_1_; CI, confidence interval.

**Figure 4:**
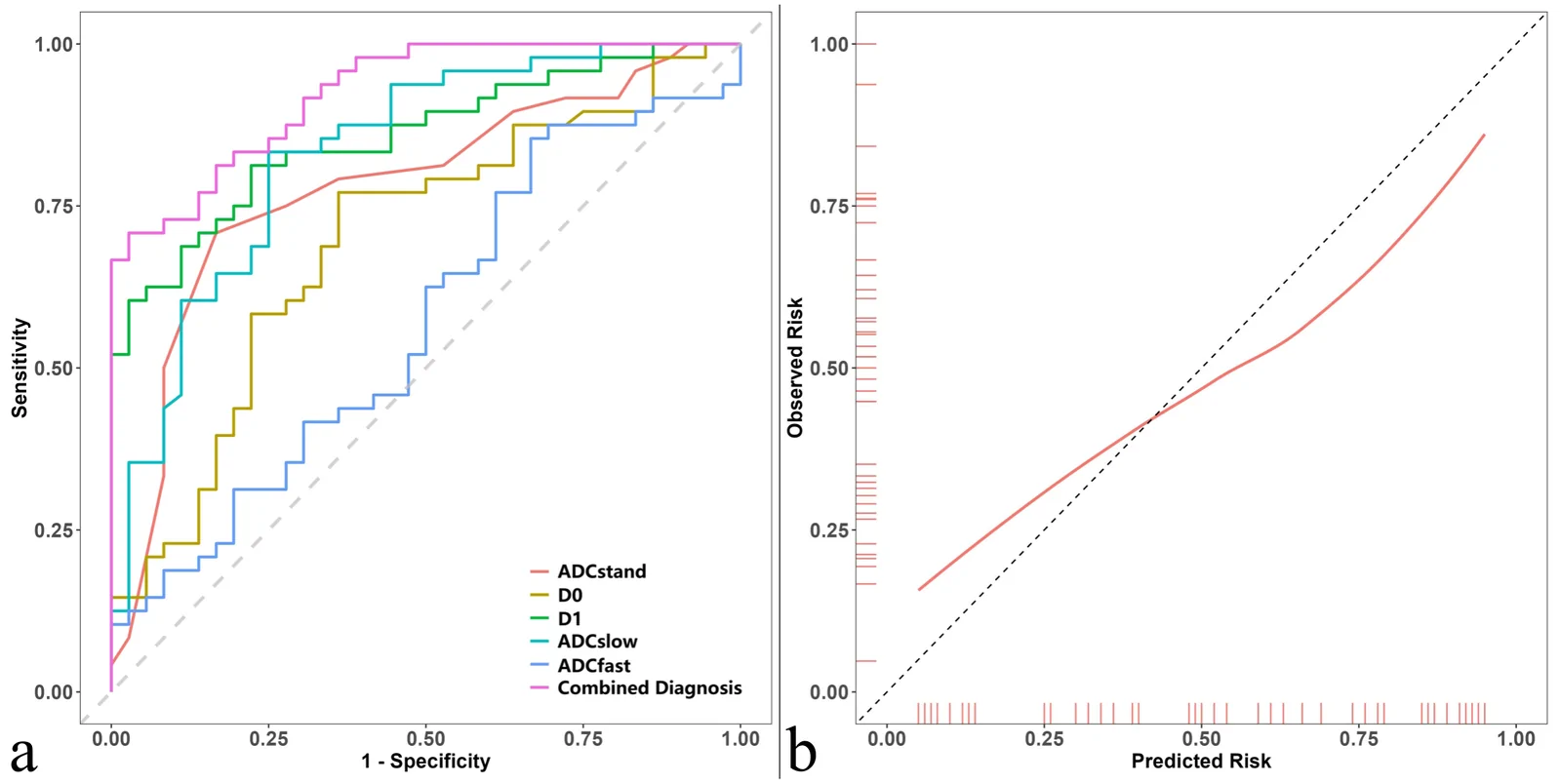
Diagnostic performance of different parameters and predictive models: **(a)** Receiver operating characteristic (ROC) curves of individual parameters and the combined predictive model (D_1_ + ADC_slow_); **(b)** Calibration curve of the combined predictive model (D_1_ + ADC_slow_), indicating good consistency between predicted and actual proliferation status.

**Figure 5:**
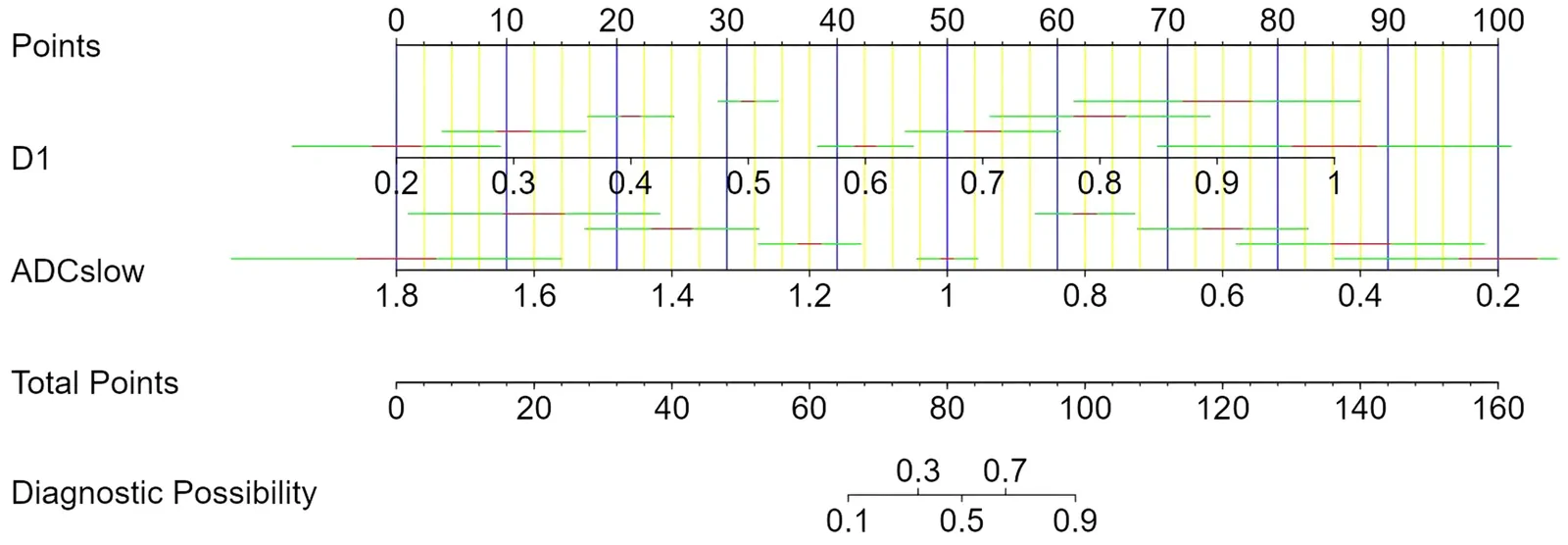
Nomogram for predicting the proliferation status of rectal carcinoma, constructed based on the independent predictors (D_1_ + ADC_slow_).

## Discussion

### Principal findings

The findings of this study indicate that quantitative parameters derived from the conventional monoexponential DWI, IVIM, and VDC diffusion signal models can effectively distinguish high-proliferation from low-proliferation rectal carcinoma. Among these parameters, ADCslow and D_1_ were identified as independent predictors of high Ki-67 expression, and the combined diagnostic model integrating the two parameters achieved the best diagnostic efficacy, with good stability verified by calibration curve analysis.

### Performance of conventional DWI

As a classical functional MRI technique, DWI detects the random Brownian motion of water molecules in tissues based on the Gaussian diffusion assumption (24). The ADC value, as the core quantitative parameter of DWI, can indirectly reflect the density of tumor cells and the characteristics of the tissue microenvironment. Previous studies have confirmed that tumor lesions with high Ki-67 expression have more active cell proliferation, higher cell density, and reduced extracellular space, which restrict the free diffusion of water molecules and thus lead to decreased ADC values (7, 25). In the present study, we observed significantly lower ADC values in the high-proliferation group compared with the low-proliferation group, which is consistent with previous research findings on rectal carcinoma and other solid tumors, further supporting the value of conventional DWI in noninvasive evaluation of tumor proliferative activity.

### Performance of IVIM-derived parameters

IVIM is an advanced multi-b-value diffusion imaging technique developed on the basis of conventional DWI. Through bi-exponential fitting of the DWI signal attenuation curve, IVIM can separate the true diffusion component of tissue water molecules from the pseudo-diffusion component caused by microcirculatory perfusion, providing more detailed tissue microstructure information (10). As a parameter reflecting pure water molecule diffusion in the IVIM model, ADC_slow_ eliminates the interference of microcirculatory perfusion compared with conventional ADC, and thus can more accurately reflect the actual diffusion restriction of water molecules caused by increased cell density (26). In this study, both ADC and ADC_slow_ showed significant differences between the two proliferation groups, but ADC_slow_ had higher diagnostic efficacy, which confirms the advantage of removing perfusion confounding factors in the evaluation of tumor cell density. The underlying mechanism is that high-proliferation rectal carcinoma has faster cell division, higher cell density, and narrower intercellular spaces, which further limits the free diffusion of water molecules, resulting in a significant decrease in ADC_slow_ values (14, 15). This result is consistent with previous studies on colorectal cancer, indicating that ADC_slow_ has important reference value for evaluating the proliferative activity of rectal carcinoma.

The perfusion fraction f is another key parameter of the IVIM model, which mainly reflects the proportion of microvascular perfusion in the voxel and is positively correlated with tumor microvessel density (15). Previous studies on bone tumors and sinonasal malignancies have reported a positive correlation between f value and Ki-67 expression level (27, 28). In our study, the f value in the high-proliferation group was significantly higher than that in the low-proliferation group, which is consistent with the above findings. The possible mechanism is that rapidly proliferating tumor tissues require more blood supply to support their growth, leading to increased microvessel density and more active microcirculatory perfusion, which is manifested as an elevated f value (29, 30). As for ADC_fast_, another perfusion-related parameter in the IVIM model, it mainly reflects the velocity of blood flow in microvessels. Most existing studies have shown that the measurement of ADC_fast_ has poor stability and reproducibility, which may be related to factors such as respiratory motion artifacts and intestinal gas artifacts in pelvic scanning. In this study, no statistically significant difference in ADCfast was found between the two proliferation groups, which is consistent with most previous clinical studies. This suggests that the clinical application value of ADC_fast_ in rectal carcinoma still needs to be further explored and verified with larger sample sizes.

### Performance of VDC-derived parameters

VDC is a newly proposed non-Gaussian diffusion imaging model in recent years. Its core theoretical basis is that the apparent diffusion coefficient of tissue is not a constant value, but shows a decreasing trend with the increase of b-value, especially in the high b-value range. This change can be described by the formula D_b_ = D_0_ × exp(-b × D_1_), and the corresponding signal attenuation formula is S_b_/S_0_ = exp[-(D_0_/D_1_) × (1 - exp(-b × D1))]. In this model, D_0_ has a similar physiological meaning to conventional ADC, reflecting the overall diffusion level of water molecules, while D_1_ characterizes the curvature of ADC decay with b-value, which can reflect the complexity of tissue microstructure (20, 31). Therefore, the VDC model can simultaneously provide information on tissue cellular density and microstructural complexity, which is helpful for a more comprehensive characterization of tumor biological behavior. A previous study by Zhang et al. applied VDC imaging to the evaluation of microvascular invasion in hepatocellular carcinoma, and found that D_1_ value was significantly higher in lesions with microvascular invasion (17). The present study is the first to explore the application value of VDC in evaluating Ki-67 expression in rectal carcinoma. Our results showed that the high-proliferation group had higher D_1_ values and lower D_0_ values compared with the low-proliferation group, and D_1_ was an independent predictor of high proliferative activity. The underlying pathological basis may be that rectal carcinoma with high Ki-67 expression has more active cell proliferation, denser cell arrangement, and more complex tissue microstructure, which leads to a faster decay rate of ADC with b-value, manifested as increased D_1_ and decreased D_0_ (32, 33).

There are still some limitations in this study that need to be acknowledged. First, this is a single-center retrospective study with a limited sample size, which may have potential selection bias. Multi-center studies with larger sample sizes are needed in the future to further verify the reliability of our findings. Second, as an emerging diffusion imaging technique, VDC still has relatively few clinical application studies at present, and the exact pathophysiological significance of its derived parameters D_0_ and D_1_ in rectal carcinoma needs to be further elucidated by combining more pathological and molecular studies. Third, the multi-b-value DWI sequence used in this study adopted the single-shot EPI acquisition method, which may have certain image distortion and artifacts for some small lesions, affecting the accuracy of measurement. In future studies, the application of advanced sequences such as readout-segmented EPI may help to improve image quality and measurement accuracy.

## Conclusion

In summary, this study highlights the potential of the VDC model for noninvasive evaluation of Ki-67 expression in rectal carcinoma. The VDC-derived parameter D_1_ emerged as a promising diagnostic biomarker, and its combination with the IVIM-derived ADC_slow_ achieved the highest predictive accuracy for identifying high-proliferation rectal carcinoma. Given the prognostic significance of Ki-67 in rectal carcinoma, this predictive model may provide valuable insights for clinical decision-making and individualized treatment planning.

## Data Availability

The raw data supporting the conclusions of this article will be made available by the authors, without undue reservation.
